# Exploring the relationship between patients’ information preference style and knowledge acquisition process in a computerized patient decision aid randomized controlled trial

**DOI:** 10.1186/s12911-015-0168-0

**Published:** 2015-06-19

**Authors:** Anna M. Sawka, Sharon Straus, Gary Rodin, Richard W. Tsang, James D. Brierley, Lorne Rotstein, Phillip Segal, Amiram Gafni, Shereen Ezzat, David P. Goldstein

**Affiliations:** Division of Endocrinology, Toronto General Hospital, 200 Elizabeth Street, 12 EN-212, Toronto, ON M5G 2C4 Canada; Department of Medicine, St. Michael’s Hospital, 30 Bond Street, Shuter 2-026, Toronto, ON M5B 1W8 Canada; Keenan Research Centre of the Li Ka Shing Knowledge Institute, University of Toronto, Toronto, ON M5B 1W8 Canada; Department of Psychosocial Oncology, Princess Margaret Hospital, University Health Network, 16th Floor Room 724, 610 University Avenue, Toronto, ON M5G 2M9 Canada; Department of Psychiatry and Palliative Care, Princess Margaret Hospital, University of Toronto, 16th Floor Room 724, 610 University Avenue, Toronto, ON M5G 2M9 Canada; Department of Radiation Oncology, Princess Margaret Hospital, University Health Network, 5th Floor Room 963, 610 University Avenue, Toronto, ON M5G 2M9 Canada; Department of Surgery, Toronto General Hospital, University Health Network, 200 Elizabeth Street, 10 EN-220, Toronto, ON M5G 2C4 Canada; Toronto General Hospital, University of Toronto, 200 Elizabeth Street, 10 EN-220, Toronto, ON M5G 2C4 Canada; Division of Endocrinology, Department of Medicine, Toronto General Hospital, University Health Network, 200 Elizabeth Street, 12 EN-216, Toronto, ON M5G 2C4 Canada; Toronto General Hospital, University of Toronto, 200 Elizabeth Street, 12 EN-216, Toronto, ON M5G 2C4 Canada; Department of Clinical Epidemiology and Biostatistics, McMaster University, 1280 Main Street West, CRL-208, Hamilton, ON L8S 4K1 Canada; Department of Otolaryngology Head and Neck Surgery, Wharton Head and Neck Centre, University Health Network, 3-952, 610 University Avenue, Toronto, ON M5G 2M9 Canada; Wharton Head and Neck Centre, University of Toronto, 3-952, 610 University Avenue, Toronto, ON M5G 2M9 Canada; Endocrine Oncology Site Group, University Health Network, Toronto General Hospital, Endocrine Oncology 585 University Avenue, 9NU-986, Toronto, ON M5G 2N2 Canada

**Keywords:** Cancer, Patient decision aid, Behaviour, Health information, Decision making, Consumer health information, Information seeking behaviors

## Abstract

**Background:**

We have shown in a randomized controlled trial that a computerized patient decision aid (P-DA) improves medical knowledge and reduces decisional conflict, in early stage papillary thyroid cancer patients considering adjuvant radioactive iodine treatment. Our objectives were to examine the relationship between participants’ baseline information preference style and the following: 1) quantity of detailed information obtained within the P-DA, and 2) medical knowledge.

**Methods:**

We randomized participants to exposure to a one-time viewing of a computerized P-DA (with usual care) or usual care alone. In pre-planned secondary analyses, we examined the relationship between information preference style (Miller Behavioural Style Scale, including respective monitoring [information seeking preference] and blunting [information avoidance preference] subscale scores) and the following: 1) the quantity of detailed information obtained from the P-DA (number of supplemental information clicks), and 2) medical knowledge. Spearman correlation values were calculated to quantify relationships, in the entire study population and respective study arms.

**Results:**

In the 37 P-DA users, high monitoring information preference was moderately positively correlated with higher frequency of detailed information acquisition in the P-DA (*r* = 0.414, *p* = 0.011). The monitoring subscale score weakly correlated with increased medical knowledge in the entire study population (*r* = 0.268, *p* = 0.021, *N* = 74), but not in the respective study arms. There were no significant associations with the blunting subscale score.

**Conclusions:**

Individual variability in information preferences may affect the process of information acquisition from computerized P-DA’s. More research is needed to understand how individual information preferences may impact medical knowledge acquisition and decision-making.

**Electronic supplementary material:**

The online version of this article (doi:10.1186/s12911-015-0168-0) contains supplementary material, which is available to authorized users.

## Background

Patient decision aids (P-DAs) inform patients about healthcare choices and they have been shown to improve patients’ knowledge of medical choices, accuracy of expectations of risks and benefits of choices, and participation in decision-making [1]. A large number of P-DAs, in such forms as computerized programs, brochures, decision boards, videotapes, and others, are available for use in cancer care. [2]. Furthermore, the use of cancer care P-DAs is associated with improved medical knowledge and reduced decisional conflict [2].

The International Patient Decision Aid Standards Collaboration has developed quality evaluation criteria for P-DAs, and such criteria encompass informational content, development process, and effectiveness in ensuring that decision-making is informed and values-based [3]. Such quality criteria include standardization of informational content, including presentation of numerical descriptions of event rates and probabilities of outcomes [3]. Internet-based P-DAs are a contemporary development in the field [4], and of particular interest to this study. An international, multidisciplinary panel of scientists recently reported on theoretical literature and empirical evidence the from a variety of disciplines supporting internet-based P-DAs, including the Health Belief Model, Social Cognitive Theory, Elaboration Likelihood Model, Theory of Goal Setting and Performance, and Stages of Change Theory [4]. This group of experts recommended more research be performed using internet-based P-DAs, examining issues such as interactivity of users [4].

In searching for health information on the internet, it is known that individuals’ personal information preferences impact searching behavior [5]. Furthermore, there is some research suggesting that matching information messages to patients’ information preference increases positive health behaviour, such as screening mammography [6, 7]. A need for more research in matching the way information is presented to individual patient preferences (‘patient-match’) has been called for [8]. In the area of internet-based P-DAs, the relationship between users’ information-seeking style and quantity of information obtained from P-DAs (i.e. interactivity of user traits with such programs) and resultant knowledge acquisition, is not known. Such research is critical in developing an understanding of whether personalization of internet-based P-DAs to the information preferences of the user, may be potentially beneficial.

We recently developed and tested a patient-directed computerized (internet-based) P-DA, explaining the choice of adjuvant radioactive iodine (RAI) or no RAI, for early stage papillary thyroid cancer [9–13]. In a randomized controlled trial comparing the use of this P-DA (with usual care) to usual care alone, we found that one time P-DA exposure was associated with a significant improvement of medical knowledge and reduction in decisional conflict, compared to no P-DA exposure [13].

The level of informational detail desired by thyroid cancer patients in medical decision-making is highly variable, with some individuals preferring detailed (including numerical probability) information, and others strongly preferring to avoid such details (particularly “the numbers”) [14]. Such variability may be due to differences in general information preference style in response to health threats. Miller [15–17] has designated two main information preference styles in potentially threatening circumstances: monitoring (seeking out and attending to threatening information), and blunting (avoiding threatening information and cognitively distracting from it).

We have previously reported, in a qualitative study, that thyroid cancer survivors indicated a need for a P-DA, explaining the choice of accepting or rejecting adjuvant radioactive iodine treatment, and the medium of choice for such was a computerized (internet-based) P-DA, with the ability to print the informational content [14]. As we understood from our prior research [14], that the potential users of a thyroid cancer adjuvant radioactive iodine treatment P-DA, expressed strong, variable, individual preferences for level of detail of relevant information, we developed a computerized P-DA that provides patients with the option of accessing textboxes containing supplemental detailed (largely numerical) information, or, alternatively, avoiding such details (by not clicking on the relevant textboxes). The rationale for this approach was to allow users’ who preferred to view detailed, largely numerical, information to easily access it by clicking on a link; furthermore, we enabled users who preferred to avoid such information, the option of simply not clicking on the related detailed information links. In this way, we planned for our P-DA to respect users’ autonomy, in deciding what level of detail each individual wanted to access, in the decision-making process.

In this pre-planned secondary analysis, we examined the relationship between patients’ information preference style and the following: 1) the number of times supplemental detailed information was obtained by P-DA users (i.e. the number of clicks on supplemental information within text boxes), and 2) medical knowledge. In a post-hoc secondary analysis that was suggested by a reviewer, we also examined the relationship between patients’ information preference style and decisional conflict. Our primary hypothesis was that DA users with higher monitoring information preference style would more frequently access the links to additional detailed information. It is important to note that randomization in this trial was not stratified by patients’ post-randomization information preferences, and so all secondary analyses presented herein should be considered hypothesis-generating.

## Methods

### Trial design

**Fig. 1:**
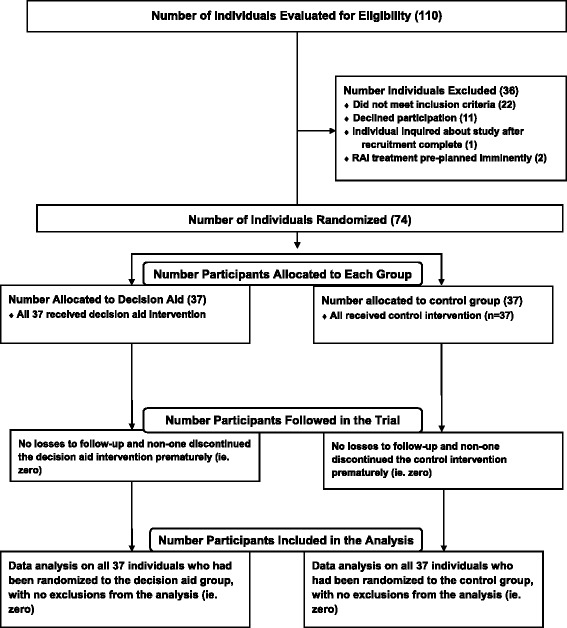
CONSORT flow diagram

We conducted a single-center parallel design randomized controlled trial, and allocated 74 consenting adults with early stage PTC in 1:1 fashion, using central computerized randomization, to either a one-time exposure to a patient-directed computerized P-DA, or no P-DA [Clinical Trials.gov identifier NCT01083550 and (12;13)] (Fig. 1). CONSORT guidelines for trial reporting were followed [18] (see Additional file 1). Blinding of patients, their physicians, and study and study personnel after allocation was not possible. The statistical analyses were un-blinded for secondary outcomes reported in this manuscript. The computerized randomization sequence incorporated random block sizes of 2 and 4 and was developed by an off-site study statistician [13]. The randomization allocation was revealed to each participant using the DA program, by a research staff member, immediately prior to the intervention allocation. The development of the P-DA included, qualitative input from patient participants on relevant information needs [14], execution of multiple relevant systematic reviews [19–23], and two phases of usability testing of respective prototypes of the P-DA (including feedback from patients, and physicians) [9–11]. This trial was approved by the University Health Network Research Ethics Board and all participants provided written, informed consent. As previously reported [13], the inclusion criteria were: individuals aged ≥18 years, who had a total thyroidectomy (completed in one or two stages) on or after September 1, 2009, and whose clinic-pathologic diagnosis was low risk papillary thyroid cancer (PTC) (i.e. primary tumor 1 to 4 cm in diameter, with no distant nor nodal metastases, no extra-thyroidal extension of the PTC, no vascular nor lymphatic invasion, and with no tall cell histologic features). Participants were required to be fluent in English (written and verbal, ascertained by self report), physically able to use a computer (by self-report), and provide informed consent [13]. We did not pre-test potential participants on their literacy or numeracy level nor level of computer proficiency, prior to enrollment in the trial. Participants in the P-DA group self-navigated the P-DA website for up to 60 min at one sitting, on a PC desktop computer, located in a research office at University Health Network. A research assistant who was present in the room, directed each individual to review a set of general instructions at the beginning of the P-DA program (on the first webpage), explaining how to navigate the program, access supplemental information, adjust font size, and print content. However, the research staff member did not assist the participants with navigation of the P-DA, nor any other procedures. P-DA users were required to visit the 16 web pages within the P-DA, but provided the option to access supplemental detailed information, presented within text boxes, labelled with an “i” and the title “More Information.” There was no limit on the number of times that P-DA group participants could access supplemental detailed information during the study visit, but self-navigation time was limited to a maximum of 60 min. No P-DA access was available outside the study. All participants received usual counselling and care from their treating physicians, but there was no standardization of the verbal consultations nor supplemental materials (eg. brochures) associated with usual care visits. All participants had been seen by at least one thyroid cancer specialist on at least one occasion, prior to viewing the P-DA. All participants were subsequently clinically followed by thyroid cancer specialists.

### Outcomes

The results of the trial primary outcome analysis, comparing medical knowledge between P-DA and no P-DA groups, has been previously reported [13]. The sample size justification of 74 participants has been previously described, for the primary outcome analysis [13], and was used as a convenience sample for all secondary analyses. The post-randomization (study baseline) information preference style was measured by respective monitoring and blunting subscale scores of the Miller Behavioural Style Scale (MBSS) [15, 16], prior to any exposure to the P-DA (or no P-DA in the case of the control group, i.e. at a concurrent time point in the study, relative to recruitment). The MBSS is a 32-item tool in which 4 hypothetical non-medical scenarios are presented (including dental work, a terrorist hostage-taking, a potential imminent employment termination, and in-flight airplane problem), followed by choices that may be categorized as either monitoring (16 choices) or blunting (16 choices) style responses; the subscale scores for monitoring and blunting are respectively calculated by summing the number of positive responses in each category (maximum score of 16 for each category) [15, 16]. The quantity of supplemental detailed medical information actively sought by P-DA users was measured by the number of clicks on text boxes titled “More Information.” There were a total of 21 such text boxes located throughout the P-DA, and when a participant clicked on one of these text boxes or icons, an event was identified. Multiple clicks on the same text box at separate discrete time points were individually counted toward the total number of clicks (for participants who returned to a textbox after closing it). The data on number of clicks for more information was collected within the P-DA. Detailed numerical information was included in such text boxes, but not all of the detailed information was necessarily numerical. Medical knowledge was assessed using a previously validated 10-item true/false questionnaire, focused on early stage PTC and information relevant to the choice to accept or reject adjuvant radioactive iodine treatment (score represented by the total number of correct responses) [9, 10]. The medical knowledge questionnaire includes 10 questions (true or false) on the following topics: low PTC prognosis, radioactive iodine treatment procedure (including preparation), potential radioactive treatment side effects, medical follow-up implications of radioactive iodine treatment, and the best available medical evidence on the impact of radioactive iodine treatment on long-term thyroid cancer outcomes [9, 10]. The knowledge questionnaire was scored by the number of correct responses (maximum possible score of 10 out of 10) [9, 10]. Decisional conflict on the decision to accept or reject radioactive iodine treatment was measured using a previously validated questionnaire [24, 25]. All questionnaires were self-administered by the participants, using pen and paper, under the supervision of a research assistant.

### Statistical methods

Data were analyzed in an intention-to-treat fashion. Descriptive data were summarized using numbers and percentages; continuous data were summarized using means and standard deviations (SD, or ranges). An alpha level of 0.05 was the cut-off for statistical significance for all analyses. In these pre-planned secondary analyses, we examined Spearman’s correlations (r) between respective baseline monitoring and blunting information preference style subscale measurements (using the Miller Monitor-Blunter Style Scale) and a) the amount of supplemental detailed information obtained in the P-DA program (measured by the number of clicks for more information), as well as b) score on a medical knowledge questionnaire in the entire study population, as well as in the respective P-DA and control groups. A post-hoc correlation analysis was performed, examining the relationship between respective baseline monitoring and blunting information preference style subscale measurements (using the Miller Monitor-Blunter Style Scale) and decisional conflict, as per recommendation of a reviewer. We also performed a post-hoc exploratory Spearman’s correlation analysis examining the relationship between the amount of supplemental detailed information obtained and medical knowledge. The rationale for this post-hoc analysis was to explore whether there was a relationship between the amount of informational content obtained by participants and ultimate success in knowledge acquisition (i.e. to understand if obtaining more detailed information related to more knowledge). Quantitative statistical analyses were performed using PASW Statistics 18.0 (IBM, Chicago, IL).

## Results

### Participant characteristics

There were 74 study participants recruited, including 37 who were exposed to the P-DA, and 37 controls, who were not exposed to the P-DA. The details of the demographic and disease characteristics of the study population have been previously reported [13]. As reflected in Table 1, the respective P-DA and control groups were similar in terms of gender, age, level of education, and frequency of computer use. All 37 participants in the P-DA group were able to complete the viewing of the 16 web pages within the allotted time (maximum 60 min). As previously reported [13], the mean time spent viewing the P-DA was about 30 min, and ranged from 11 to 60 min, and none of the participants reported any major difficulties navigating the site. None of the participants in the control group viewed the DA at any time point before, during, or after the study. There was no harm reported to participants in this study.

**Table 1:** Participant characteristics

Characteristic		Patient decision aid group (*n* = 37)	Control group (*n* = 37)
Females		31 (84 %)	31 (84 %)
Mean Age in Years (Standard Deviation)		48 (12)	44 (12)
Highest Education Attained	High School or Lower	4 (11 %)	5 (14 %)
	College or University	33 (89 %)	32 (86 %)
Self-report Frequency of Computer Use	Utilize computer most days	34 (92 %)	36 (98 %)
Miller Behavioural Style Scale Scores	Mean Monitoring Subscale Score (Standard Deviation)	10.9 (2.8)	10.2 (2.6)
	Mean Blunting Subscale Score (Standard Deviation)	4.0 (2.4)	4.1 (2.3)

Measured using the Miller Behavioural Style Scale (maximum value for each measure = 16)

The Miller Monitoring subscale result for the entire study population was a mean of 10.6 (out of a maximum of 16), standard deviation 2.7, minimum 3, maximum 16 (data from 74 individuals). The Miller Blunting subcale result for the entire study population was a mean score of 4.0 (out of a maximum of 16), standard deviation 2.3, minimum 0, maximum 10 (data from 74 individuals). For the entire study population of 74 individuals, the mean medical knowledge score was 8.8 (out of a maximum of 10), standard deviation 1.4, minimum of 4, and maximum of 10. For the entire study population (*n* = 74), the mean decisional conflict score (out of a maximum of 100) was 38.6, standard deviation 22.5, minimum 2, maximum 98. As previously reported [13], the mean medical knowledge score in the P-DA group was 9.7 out of 10 (standard deviation 0.6, from *n* = 37 participants), which was significantly higher than the mean score of 7.8 (standard deviation 1.3 from *n* = 37 participants) in the control group (*p* < 0.001 for the comparison). Furthermore, as previously reported [13], decisional conflict score was significantly lower in the P-DA group (mean score of 25.2, standard deviation 13.4 from *n* = 37 participants) compared to the control group (mean score of 52.1, standard deviation 21.9 from *n* = 37 participants) (*p* < 0.001 for the difference). The Miller Monitor subscale results according to randomization status were as follows: P-DA group, mean score (out of a maximum of 16) was 10.9, standard deviation 2.8, minimum 3, maximum 16, from 37 individuals, control group mean score 10.2, standard deviation 2.3, minimum 5, maximum 15, from 37 individuals. The Miller Blunting subscale results according to randomization status were as follows: P-DA group, mean score (out of a maximum of 16) was 4.0, standard deviation 2.4, minimum 1, maximum 10, from 37 individuals, control group mean score 4.1, standard deviation 2.3, minimum 0, maximum 9, from 37 individuals. For the 37 individuals in the P-DA group, the mean number of clicks to obtain “More Information” was 11.1 (standard deviation 10.9, range from 0 to 41).

### Information preference style and supplemental P-DA data obtained

Our first objective was to determine whether there was a relationship between participants’ information preference style and the quantity of supplemental information obtained in the P-DA program by clicking for “More Information”. We found a significant moderate positive correlation between the monitoring (higher information seeking preference) subscale score and the quantity of supplemental P-DA data obtained using “More Information” clicks (*r* = 0.414, *p* = 0.011). However, there was no significant relationship between the blunting (information avoidance preference) subscale score and the number of clicks for supplemental information (*r* = 0.117, *p* = 0.489) in the P-DA group (*n* = 37).

### Information preference style and medical knowledge acquired

A second objective was to determine whether there was a relationship between the individual information preference style and medical knowledge, for the entire study population, as well as the intervention and control subgroups, respectively. For the entire study population of 74 individuals, the monitoring subscale score was significantly, weakly positively correlated with the number of correct responses on a thyroid cancer medical knowledge questionnaire (*r* = 0.268, *p* = 0.021); however, the blunting subscale score was not associated with the medical knowledge score (*r* = −0.124, *p* = 0.292). For the 37 individuals in the P-DA group, there was no statistically significant association between monitoring subscale score and number of correct responses on the medical knowledge questionnaire (*r* = 0.277, *p* = 0.097), but the relatively small size of this subgroup likely limited statistical power of this analysis, given the observed trend. Furthermore, the blunting subscale score was not associated with medical knowledge questionnaire score (*r* = 0.031, *p* = 0.855) in P-DA users. For the 37 individuals in the control group, medical knowledge was also not significantly associated with either the monitoring subscale score (*r* = 0.221, *p* = 0.189), nor the blunting subscale score (*r* = −0.246, *p* = 0.143).

### Post-hoc exploratory analysis examining information preference style and decisional conflict

In a post-hoc exploratory statistical analysis suggested by a reviewer, we examined whether information preference style was associated with decisional conflict in the entire study population as well as in the respective subgroups (P-DA and control). Decisional conflict was measured post-P-DA intervention in the P-DA group. We found no statistically significant relationships between decisional conflict and the Miller Monitoring subscale score in the entire study population (*r* = −0.020, *p* = 0.866, *n* = 74), P-DA group (*r* = −0.029, *p* = 0.867, *n* = 37), nor in the control group (*r* = 0.127,*p* = 0.454, *n* = 37). Furthermore, we found no statistically significant relationships between decisional conflict and the Miller Blunting subscale score in the entire study population (*r* = 0.002, *p* = 0.987, *n* = 74), P-DA group (*r* = 0.278, *p* = 0.096, *n* = 37), nor control group (*r* = −0.227, *p* = 0.176, *n* = 37).

### Post-hoc exploratory analysis examining quantity of supplemental information obtained and medical knowledge

In a post-hoc exploratory statistical analysis in the P-DA group, there was no relationship between the number of clicks for supplemental detailed information and the medical knowledge score (*r* = −0.154, *p* = 0.361) (*n* = 37). Although statistical power of this analysis was limited, the inverse relationship suggested that accessing supplemental detailed information was unlikely to be a major contributor to medical knowledge.

## Discussion and conclusions

We observed a high degree of variability among thyroid cancer patients, in the quantity of additional detailed information sought within a computerized P-DA on RAI decision-making. We also observed that that high monitoring information preference style was associated with increased frequency of obtaining such supplemental from the P-DA. These findings are consistent with results of prior studies of oncology patients, indicating that high monitoring style is associated with general preference for more detailed medical information [26] and, in the case of oncologic healthcare consultations, increased number of questions posed by the patient [26, 27]. High monitoring subscale score on the Miller Behavioural Style questionnaire has also been associated with increased utilization of specialized sources of information by breast cancer patients, particularly relating to medical books or journals [28]. We did not observe a significant relationship between blunting style and information obtained from the P-DA. In previous studies, Ong et al. reported that a blunting style was not correlated with desired level of detail of cancer-related information [26], whereas Timmermans et al. reported that higher blunting subscale scores were significantly associated with fewer questions posed by patients during palliative radiotherapy consultations, but not curative radiotherapy consultations [27]. Differences in the tools used to measure blunting or differences in study populations, could potentially explain the variability in these findings. We did not find convincing evidence to suggest that increased rate of obtaining of detailed information improved medical knowledge scores. Our results also fail to support the assumed (or intuitive) model that a preference to seek more information (high monitoring style) leads to significantly more medical knowledge in computerized P-DA users. More future research is needed, ideally in larger studies, to verify our findings and better understand the potential clinical impact.

The strengths of this study include: its novelty as the first study of computerized P-DAs exploring the quantity of information sought by users as well as the relationship between individual information preferences and the process of information acquisition and ultimate knowledge, the standardization to the procedure in which the P-DA was presented (i.e. in a research office, timed use, no exposure to the P-DA intervention before or after the session), complete data collection (i.e. no missing data), the automated recording of the number of clicks for supplemental information obtained within the P-DA by participants, and the utilization of a well-established, validated tool for measurement of information preference style. Some of the limitations include the following: this is a secondary analysis and the randomization was not stratified according to information preference (so the trial was not specifically designed to primarily address whether information preference impacts other outcomes and as such, results are hypothesis-generating), the sample size was relatively small so the secondary analyses (including subgroup analyses) may have been statistically underpowered, potential selection bias (most participants reported using computers on most days and most were well-educated, further all were fluent in written and spoken English), lack of standardization of the counseling (or any related materials) received by treating physicians as part of usual care, and limited external generalizability to healthcare decisions or other computerized P-DAs where users do not have the option to choose to view additional information or not.

In conclusion, our study demonstrates the variability that exists among thyroid cancer patients, in terms of individual information seeking preferences, and resultant quantity of detailed information retrieved from a computerized P-DA. Although hypothesis-generating, our results also raise some questions about the incremental value of mandating presentation of detailed numerical data within P-DA’s, as some patients may prefer not to view such data, and it is not clear that those who view it, acquire more fundamental medical knowledge (as compared to a simpler explanation). An important implication of our findings for developers of P-DAs and researchers, is the introduction of the concept of personalizing knowledge translation to the information preferences of users. Internet-based P-DAs may be particularly amenable to personalized modifications, allowing users to navigate links, based on information preferences. Yet, to some extent, tailoring informational detail in decision support tools by allowing some users to avoid detailed numerical probability information according to their preferences could be at odds with current P-DA quality standardization initiatives [3]. Future studies are needed, to explore whether personalization of format and the level of detail of content (particularly relating to numerical data) of P-DAs to users’ preferences may enable optimization of knowledge translation outcomes, or at least be non-inferior to current methods, but potentially preferred by some users. Such research is critical in advancing our understanding of the evolving role of personalization of decision support tools in advancing patient autonomy regarding provision of information in medical decision-making.

## Additional file

Additional file 1:
**CONSORT 2010 checklist of information to include when reporting a randomised trial*.**
